# LncRNA linc00152/NF‐κB feedback loop promotes fibroblast‐like synovial cells inflammation in rheumatoid arthritis via regulating miR‐103a/TAK1 axis and YY1 expression

**DOI:** 10.1002/iid3.417

**Published:** 2021-06-01

**Authors:** Jian Zhang, Fei‐Fei Gao, Jie Xie

**Affiliations:** ^1^ Department of Rheumatology and Immunology Weihai Municipal Hospital Weihai Shandong China; ^2^ Department of Out‐Patient Weihai Municipal Hospital Weihai Shandong China

**Keywords:** fibroblast‐like synoviocytes, inflammation, linc00152, rheumatoid arthritis

## Abstract

**Introduction**: Overexpressed inflammatory cytokines are the main factors causing rheumatoid arthritis (RA) tissue damage and pathological deterioration, and lncRNAs has found to beinvolved in some autoinflammatory diseases. **Methods**: We designed this study to investigate the effect of lncRNA linc00152 on rheumatoid arthritis inflammation and explore its molecular mechanism.**Result**: We found that linc00152 was not only up‐regulated in rheumatoid arthritis fibroblast‐like synoviocytes (RAFLS), but also stimulated by TNF‐α/IL‐1β in adose‐ and time‐dependent manner in RAFLS and this expression depends on the NF‐κB signaling pathway. Conversely, linc00152 promoted TNF‐α/IL‐1β expression in RAFLS induced by TNF‐α/IL‐1β. In addition, we found that linc00152 promoted TAK1 expression by targeting inhibition of miR‐103a and activated TAK1‐mediated NF‐κB pathway. NF‐kB indirectly promotes linc00152 expression by promoting the transcription activity of YY1, and YY1 directly promotes linc00152 expression by binding the promoter of linc00152. **Conclusion**: Our data suggested that the linc00152/NF‐κB feedback loop promotes RAFLS inflammation via regulating miR‐103a/TAK1 axis and YY1 expression. Thus, linc00152 acts as a switch to control this regulatory circuit and may serve as a diagnostic and therapeutic target for RA treatment.

## Graphical abstract

linc00152 promotes inflammatory cytokines expression in RAFLS linc00152 promotes TAK1 expression by targeting miR‐103a linc00152 activates TAK1‐mediated NF‐κB pathway.

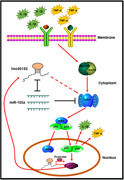

## 1 INTRODUCTION

Rheumatoid arthritis (RA) is a chronic systemic autoimmune disease characterized by synovial inflammation, and the destruction of articular cartilage in RA is closely related to the process of bone remodeling induced by synovial inflammation.^1^, ^2^ It leads to severe joint destruction and eventually functional lesions, with a very high disability rate. In recent years, many studies have shown that the excessive proliferation of RA fibroblast‐like synoviocytes (RAFLS) and the production of inflammatory factors are the keys to the development and metastasis of RA.^3^, ^4^ Fibroblast‐like synoviocytes (FLS) have a function of tissue shaping under physiological conditions and matrix remodeling during pathological injury in the joint cavity.^5^ FLS exhibits abnormal proliferation in synovial lesions of RA, and is the main effector cell that mediates joint destruction and synovial inflammation because it not only responds to a variety of inflammatory cytokines,^5^ but also secretes a series of cytokines to play a pleiotropic role on monocytes, lymphocytes and bone cells.^6^, ^7^ Therefore, studying the molecular mechanism of RAFLS inflammation regulation is very important for the treatment of RA patients.

There are 75% of human genomic DNA being transcribed into RNA, but of only 2% of the genome encodes proteins, and 98% of transcripts are noncoding RNA.^8^, ^9^ A single‐stranded RNA molecule with a length of about 20–24 nucleotides is called a noncoding single‐stranded RNA molecule, and a noncoding RNA with a length of more than 200 nucleotides is called a long‐chain noncoding RNA (lncRNA).^8^, ^9^ lncRNA was originally considered to be "junk" RNA, but in recent years research works have found that lncRNA plays an important role in many life activities such as dose compensation effect, epigenetic regulation, cell cycle regulation, and cell differentiation regulation.^10^, ^11^ In recent years, more and more lncRNAs have been found to play an important role in the occurrence and development of RA.^12^, ^13^ These lncRNAs were found to participate in the regulation of apoptosis,^14^ inflammatory factor secretion,^15^, ^16^ proliferation, and migration^13^ in RAFLS.

In the present study, we analyzed the microarray data from GEO datasets to obtain differentially expressed lncRNAs, and then lncRNA linc00152 was focused on for further study because it was not only significantly up‐expressed in RAFLS, but also was found to be involved in the regulation of RAFLS proliferation and apoptosis,^17^ and could regulate nuclear factor‐κB (NF‐κB) pathway (an important pathway was related to RAFLS).^18^ Here, we focus on the research of linc00152 on the RAFLS inflammation. We hypothesized that linc00152 was involved in the regulation of RAFLS inflammation and might be related to the NF‐κB pathway. Fortunately, our preliminary data indicated that the up‐regulation of linc00152 by tumor necrosis factor‐α/interleukin‐1β (TNF‐α/IL‐1β) was NF‐κB‐dependent, and linc00152 activated the TAK1‐mediated NF‐κB pathway by targeting inhibition of miR‐103a. Thus, linc00152 acts as a switch to control this regulatory circuit and may serve as a diagnostic and therapeutic target for RA treatment.

## 2 MATERIALS AND METHODS

### 2.1 Tissues and separate RAFLS

We collected 11 synovial tissues from RA patients, and five synovial tissues from trauma patients who were confirmed to have no bone and joint diseases. Studies related to human tissues are conducted in accordance with the research protocol approved by Weihai Municipal Hospital Ethics Committee. At the same time, we separated FLS from synovial tissue as previously described.^19^ Briefly, synovial tissue was cut into small pieces, and then added 1 mg/ml Collagenase I (Thermo Fisher Scientific) solution to digest for 6 h at 37°C in a CO_2_ incubator, and then replaced the Collagenase I solution with Dulbecco's modified Eagle's medium (Thermo Fisher Scientific) supplemented with 10% fetal bovine serum (Hyclone). After being identified by cell membrane biomarker (CD14−CD68−CD90+), the third‐to‐sixth‐generation RAFLS is used for research in the present study.

### 2.2 Real‐time quantitative polymerase chain reaction (PCR) assay

We used RNAiso plus (Takara) to extract total RNA from cells. After preparing the cDNA using a PrimeScript RT reagent Kit with gDNA eraser (Takara), 20 μl of the qPCR system was prepared and analyzed as described in the instructions of GoTaq qPCR Master Mix (Promega). The relative expression of gene was calculated by 2^‐∆∆Ct^ method. β‐actin was loaded as control for mRNA, and U6 was loaded as control for miRNA. Primers are shown in Table 1.

**Table 1:** Sequence of primers for qPCR, siRNA, and miRNA regulation, and information of antibody

Primers used in qPCR analysis (5'‐3')		
linc00152	Forward: CTACTGCTGAGAGACCCCCT	
	Reverse: CTAACCCATGGGGACCACAC	
miR‐103a	Forward: ACACTCCAGCTGGGAGCAGCATTGTACAGGG	
	Reverse: TGGTGTCGTGGAGTCG	
TAK1	Forward: CCTCTGGCCCATTGAGTGTTT	
	Reverse: GTCCACTGCGGTGACTATCTG	
U6	Forward: AUAAAUCCCUUUACACCUCTT	
	Reverse: AAUAAAUCCCUUUACACCUCTT	
β‐actin	Forward: AGCCCATCCTTCGAGTACAAA	
	Reverse: TCTTGGTGCGATAACTGGTGG	
BS1 for CHIP	Forward: GCCTCTCCTTGGTGAGTATGTT	
	Reverse: AGCCCAGACTCCAAGGCAAA	
BS2 for CHIP	Forward: AGGACACTGCGTTTCTTTCAGA	
	Reverse: ACAGTAACCATGGGGCTGGT	
si‐RNA sequence (5'‐3')		
si‐NC	CCUGCGUUGUAAGAAUGGGAUA	
si‐00152	UCAAAGUCUGAAAGAAACGCA	
si‐YY1	UCUUUAGCUGAGUAAAUAGAU	
miRNA regulate sequence (5'−3')		
miR‐103a‐NC	AUGGGCUAACGCGGCAUCGACGA	
miR‐103a‐mimic	AGCAGCAUUGUACAGGGCUAUGA	
miR‐103a‐inhibitor	UCGUCGUAACAUGUCCCGACACU	
Information of antibody		
Name of antibody	Cas: #	Company
Anti‐MyD88 antibody	ab133739	Abcam
Anti‐TRAF6 antibody	ab33915	Abcam
Anti‐TAK1 antibody	ab109526	Abcam
Anti‐IKB alpha antibody	ab12134	Abcam
Anti‐IKB alpha (phospho S36) antibody	ab133426	Abcam
NF‐κB p65 (D14E12) antibody	8242	Cell Signaling Technology
Phospho‐NF‐κB p65 (Ser536) antibody	3033	Cell Signaling Technology
Lamin A (133A2) antibody	86846	Cell Signaling Technology
YY1 (D3D4Q) Rabbit mAb	63227	Cell Signaling Technology
Goat anti‐rabbit IgG (H + L)‐HRP	HS101‐01	TransGen Biotech
Goat anti‐mouse IgG (H + L)‐HRP	HS201‐01	TransGen Biotech
Anti‐β‐actin antibody	HS202‐01	TransGen Biotech

### 2.3 Fluorescence in situ hybridization

A number of 1 × 10^5^ cells were seeded into Lab‐Tek chambered (Thermo Fisher Scientific) to culture for 24 h at 37°C with 5% CO_2_. Next day, we removed the cell culture medium and fixed cell with 4% paraformaldehyde for 10 min at room temperature. And then cells were blocked with 5% bovine serum albumin for 1 h at room temperature. After fixing and blocking, cells were incubated with a fluorescent probe for binding to the human version of the linc00152 gene which was synthesized by Genomeditech Co., Ltd. For cells, it should be counterstained the nucleus with 5 μg/ml 4′,6‐diamidino‐2‐phenylindole for 5 min at room temperature. At last, all samples were analyzed by confocal microscopy.

### 2.4 Cell transfection

We directly transfected 50 nmol/L of siRNA into 2.5 × 10^6^ RAFLS using Lipofectamine 2000 (Thermo Fisher Scientific) according to the manufacturer's protocols. After 72 h of transfection, we performed experiments. For wild type (WT) or mutated (MUT) versions of the 3'‐UTR of linc00152 and TAK1 were cloned into pisCHECK2 (Addgene), and then began transfection into cells as siRNA, and used a Dual‐Lucy Assay Kit (Solarbio) to detect luciferase activity following the manufacturer's protocol. After 72 h, gene expression was determined by RT‐qPCR or western blot.

For overexpression of linc00152, we first cloned the gene sequence of linc00152 into pcDNA3.1 plasmid (Youbao Biotechnology Co., Ltd), and transferred the recombinant plasmid into RAFLS using Lipofectamine 2000. Seventy‐two hours later, cells were cultured with 200 μg/ml G418 for 7 days, puromycin resistant cells were set for experiments. At last, we determined linc00152 expression by RT‐qPCR.

### 2.5 Western blot

We used Nuclear/Cytosol Fractionation Kit (Biovision) to extract the nuclear protein. And we analyzed 40 μg total protein using a 10% sodium dodecyl sulfate‐polyacrylamide gel electrophoresis (SDS‐PAGE). After transfer, polyvinylidene fluoride membranes (Thermo Fisher Scientific) is first blocked with 5% skimmed milk powder, and then is probed with primary antibodies and secondary antibodies. Proteins were visualized with ECL solution (Beijing Xinjingke Biotechnologies Co., Ltd.), followed by densitometry analysis using Imag J 3.0 (IBM) and β‐actin was loading as control. All information of antibodies used for immunoblotting testing is shown in Table 1.

### 2.6 Chromatin immunoprecipitation

We used chromatin immunoprecipitation (ChIP) assay kit (Millipore) to analyze the YY1 antibody capture linc00152 promoter DNA according to the manufacturer's protocols. In brief, cells were cross‐linked chromatin using 1% formaldehyde for 10 min at 37°C, and then resuspend cell to 1 × 10^6^ cells to per 200 µl of SDS lysis buffer. Next, sonicate chromatin on ice using microultrasonic cell disruptor to shear DNA to an average fragment size of about 400–1000 bp. Add the antibody against YY1 to each sample and IP overnight with gentle rotation at 4°C. We added protein A/G PLUS agarose to adsorb the YY1 antibody of the cross‐linked DNA, and added 200 mM NaCl and incubate samples overnight at 65°C to reverse formaldehyde crosslinks. At last, we extracted DNA for PCR analysis.

### 2.7 Statistical analysis

GraphPad Prism (v8.4.2.679, GraphPad Software) was used to analyzed the data and drawing figures in this study. The difference between the two groups was analyzed by student's *t* test, and one‐way analysis of variance was used to compare the difference between multiples groups. *p* value less than .05 indicated significantly different.

## 3 RESULTS

### 3.1 linc00152 is upregulated in RAFLS

**Figure 1:**
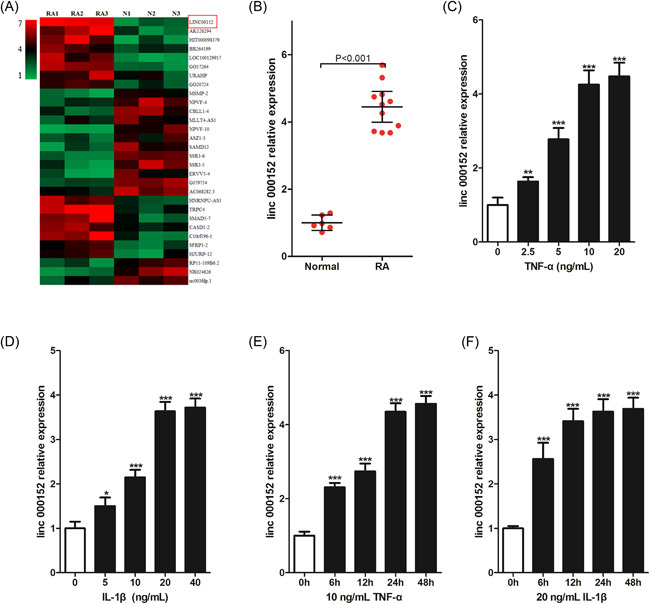
linc00152 is upregulated, and inflammatory cytokines promote linc00152 expression in RAFLS. (A) Microarray data of some upregulated or downregulated lncRNAs in fibroblast‐like synoviocytes (FLS) from three rheumatoid arthritis patients (RA) and three trauma patients (N). Data were retrieved from GEO datases, with accession no. GSE103578. (B) linc00152 was detected in FLS from 11 RA patients and five trauma patients (Normal) using RT‐qPCR. *p* value was calculated by Student's *t* test. (C–F) linc00152 was detected in rheumatoid arthritis FLS (RAFLS) (*n* = 3) after being stimulated with different dose of TNF‐α/IL‐1β for different time. *p* value was calculated by one‐way ANOVA; *p < .05, **p < .01, ***p < .001 versus control group (RAFLS without any stimulation). IL‐1β, interleukin‐1β; lncRNA, long‐chain noncoding RNA; RAFLS, rheumatoid arthritis fibroblast‐like synoviocytes; RT‐qPCR, real‐time quantitative polymerase chain reaction; TNF‐α, tumor necrosis factor‐α

According to the microarray data of lncRNAs in FLS from three patients with RA and three trauma patients (N) from GEO datasets (No. GSE103578), we identified some lncRNAs that are abnormally expressed in RAFLS, linc00152 is one of them (Figure 1A). Next, we collected 11 cases of RAFLS and five cases of normal FLS to detect the expression of linc00152, and found that linc00152 was upregulated in RAFLS (Figure 1B), which was compared with normal FLS. We hypothesized that the high expression of linc00152 in RAFLS is related to inflammatory cytokines. To test this hypothesis, we used different concentrations of TNF‐α and IL‐1β to stimulate RAFLS, and found that TNF‐α (Figure 1C) and IL‐1β (Figure 1D) induced linc00152 expression in a concentration‐dependent manner. And when the concentration of TNF‐α reached 10 ng/ml or the concentration of IL‐1β reached 20 ng/ml, the trend of increased expression of linc00152 slowed down, so we choose 10 ng/ml TNF‐α or 20 ng/ml IL‐1β for further research. Interestingly, when using a fixed concentration of TNF‐α (Figure 1E) or IL‐1β (Figure 1F) to stimulate RAFLS for different times, we found that with the prolongation of stimulation time, the expression of linc00152 gradually increased, and the growth trend slowed down at 24 h. Therefore, we determined that 10 ng/ml TNF‐α or 20 ng/ml IL‐1β stimulated RAFLS for 24 h as a condition for the next study.

### 3.2 linc00152 promotes inflammatory cytokin**e**s expression in RAFLS

**Figure 2:**
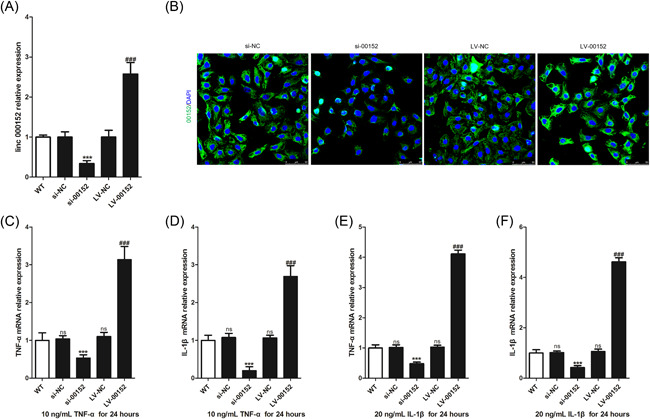
linc00152 promotes inflammatory cytokines expression in RAFLS. (A, B) 72 h after transfection of si‐NC, si‐00152, LV‐NC, and LV‐00152, we used RT‐qPCR (A) and FISH staining to determine the expression of linc00152. (C–F) TNF‐α/IL‐1β mRNA was detected in rheumatoid arthritis FLS (RAFLS) (*n* = 3) after being stimulated with 10 ng/ml TNF‐α or 20 ng/ml IL‐1β for 24 h. *p* value was calculated by one‐way ANOVA. ns was *p* > .05 versus WT group, ***p < .001 versus si‐NC group, ###*p* < .001 versus LV‐NC group. WT, wild type, indicates RAFLS without any stimulation. IL‐1β, interleukin‐1β; RAFLS, rheumatoid arthritis fibroblast‐like synoviocytes; RT‐qPCR, real‐time quantitative polymerase chain reaction; TNF‐α, tumor necrosis factor‐α

To study the effects of linc00152 in RAFLS, linc00152 overexpression or knockdown was performed to investigate its functions. First, the interfering efficiency of siRNA against linc00152 was measured by RT‐qPCR. Data suggested that si‐00152 could successfully knock down the expression of linc00152 (Figure 2A). Next, the overexpressing of efficiency of recombinant plasmid against linc00152 was measured by RT‐qPCR, and the result also showed that LV‐00152 could successfully overexpression of linc00152 (Figure 2A). Similarly, the results of FISH staining also indicated that si‐00152 knocked down and LV‐00152 overexpressed linc00152 expression in RAFLS, and also suggested that linc00152 was expressed in the cytoplasm of RAFLS (Figure 2B). We used linc00152 knockdown and linc00152 overexpression RAFLS to study its functions, and we found that after being stimulated with 10 ng/ml TNF‐α or 20 ng/ml IL‐1β for 24 h, compared with WT RAFLS, linc00152 knockdown could significantly decrease the expression of TNF‐α and IL‐1β mRNA, while linc00152 overexpression could significantly increase the expression of TNF‐α and IL‐1β mRNA in RAFLS (Figure 2C–F).

### 3.3 linc00152 promotes TAK1 expression by targeting miR‐103a

**Figure 3:**
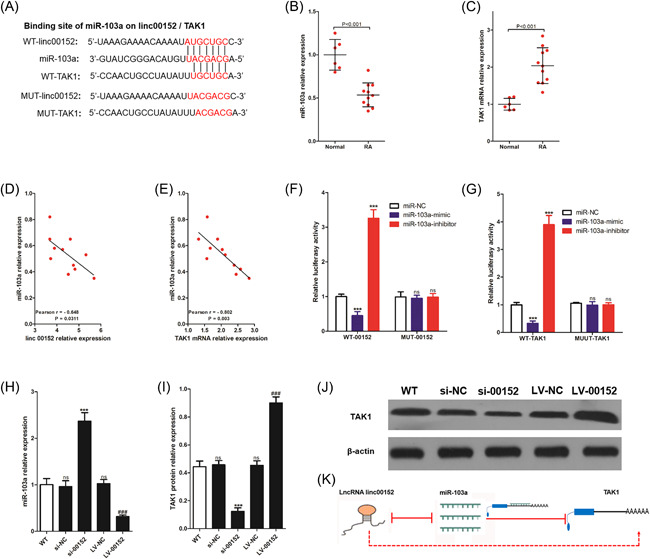
linc00152 regulates miR‐103a/TAK1 axis in RAFLS. (A) Wild‐type (WT) or mutant (MUT) sequence of linc00152 and TAK1 bound to miR‐103a. linc00152 (B) and TAK1 (C) was detected in FLS from 11 RA patients and three trauma patients (Normal) using RT‐qPCR. *p* value was calculated by Student's *t* test. Scatterplot describing the correlation between linc00152 and miR‐103a (D), TAK1 and miR‐103a (E). WT or MUT sequence of linc00152 (F) and tak (G) bound to miR‐103a were cotransferred into RAFLS with miR‐NC, miR‐103a‐mimic, or miR‐103a‐inhibitor, and then measured the luciferase activity. *p* value was calculated by one‐way ANOVA. ***p < .001 and ns was *p* > .05 versus miR‐NC group. 72 h after transfection of si‐NC, si‐00152, LV‐NC, and LV‐00152, we used RT‐qPCR to determine the expression of miR‐103a (H), and used western blot to determine TAK1 protein expression (I and J). *p* value was calculated by one‐way ANOVA. ns was *p* > .05 versus WT group, ***p < .001 versus si‐NC group, ###*p* < .001 versus LV‐NC group. WT, wild type, indicates RAFLS without any stimulation. RAFLS, rheumatoid arthritis fibroblast‐like synoviocytes; RT‐qPCR, real‐time quantitative polymerase chain reaction; TNF‐α, tumor necrosis factor‐α

To explore the mechanism of linc00152 in inflammation of RAFLS, the functions between lncRNAs and miRNAs are focused on because the ceRNA mechanism plays an important role in diseases. Next, candidates of putative targets for linc00152 were predicted by online databases (USCS, targetscan, and BiBiserv2 software). Among these, miR‐103a was chosen for predicted candidate because not only it was reported to be a RA‐related miRNA,^20^, ^21^ but also miR‐103a has reported to be bind with linc00152^22^ and has a binding site in linc00152 transcript (Figure 3A). First, we determined the expression of miR‐103a and TAK1 in 11 cases of RAFLS and five cases of normal FLS, and found that miR‐103a was downregulated in RAFLS (Figure 3C), while TAK1 mRNA was upregulated in RAFLS (Figure 3C). Additionally, we also found that there was a negative correlation between linc00152 expression and miR‐103a expression (Figure 3E), and same as miR‐103a and TAK1 in RAFLS (Figure 3E). To further study the binding mechanism, we did a luciferase gene report assay, the results have shown that miR‐103a‐mimic could significantly reduce the luciferase activity in RAFLS transferred to WT‐00152, and miR‐103a‐inhibitor could significantly increase the luciferase activity in RAFLS transferred to WT‐00152, while miR‐103a‐mimic/miR‐103a‐inhibitor could not affect the luciferase activity in RAFLS transferred to MUT‐00152 (Figure 3F). Similarly, miR‐103a‐mimic could significantly reduce the luciferase activity in RAFLS transferred to WT‐TAK1, and miR‐103a‐inhibitor could significantly increase the luciferase activity in RAFLS transferred to WT‐TAK1, while miR‐103a‐mimic/miR‐103a‐inhibitor could not affect the luciferase activity in RAFLS transferred to MUT‐TAK1 (Figure 3G). Interestingly, knocking down linc00152 upregulated the expression of miR‐103a (Figure 3H) and downregulated the expression of TAK1 protein (Figures 3I,J), but overexpressing linc00152 downregulated the expression of miR‐103a and promoted the expression of TAK1 protein. In conclusion, linc00152 promoted TAK1 protein expression by mutual inhibition with miR‐103a in RAFLS (Figure 3K).

### 3.4 linc00152 activates TAK1‐mediated NF‐κB pathway

**Figure 4:**
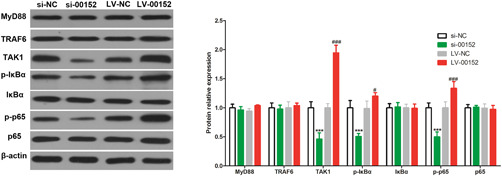
linc00152 activates the NF‐κB signaling pathway. 72 h after transfection of si‐NC, si‐00152, LV‐NC, and LV‐00152, RAFLS was stimulated by 10 ng/ml TNF‐α for 24 h, and then we used western blot to detect the expression of key protein in NF‐κB pathway. *p* value was calculated by Student's *t* test. ***p < .001 versus si‐NC group, ###*p* < .001 versus LV‐NC group. WT, wild type, indicates RAFLS without any stimulation. NF‐κB, nuclear factor‐κB; RAFLS, rheumatoid arthritis fibroblast‐like synoviocytes; TNF‐α, tumor necrosis factor‐α

Parallelly, to investigate the mechanism of linc00152 in inflammation of RAFLS, we tested the NF‐κB signaling pathway because not only NF‐κB pathway is a pathway closely related to inflammation in RA,^23^ but also TAK1, an upstream molecule of the NF‐κB pathway, can activate the NF‐κB pathway by phosphorylating IκBα via activating IKKs.^24^, ^25^ Here, 72 h after transfection of siRNAs, RAFLS was stimulated by 10 ng/ml TNF‐α for 24 h, and then we detected the expression of key proteins throughout NF‐κB pathway, and found that si‐00152/Lv‐00152 could not affect the expression of MyD88 and TRAF6 protein, two molecules located upstream of TAK1 in NF‐κB pathway, while knocking down linc00152 significantly inhibited the phosphorylation of IκBα and p65, and overexpressing linc00152 promoted the phosphorylation of IκBα and p65 (Figure 4).

### 3.5 NF‐κB pathway indirectly promotes linc00152 expression

**Figure 5:**
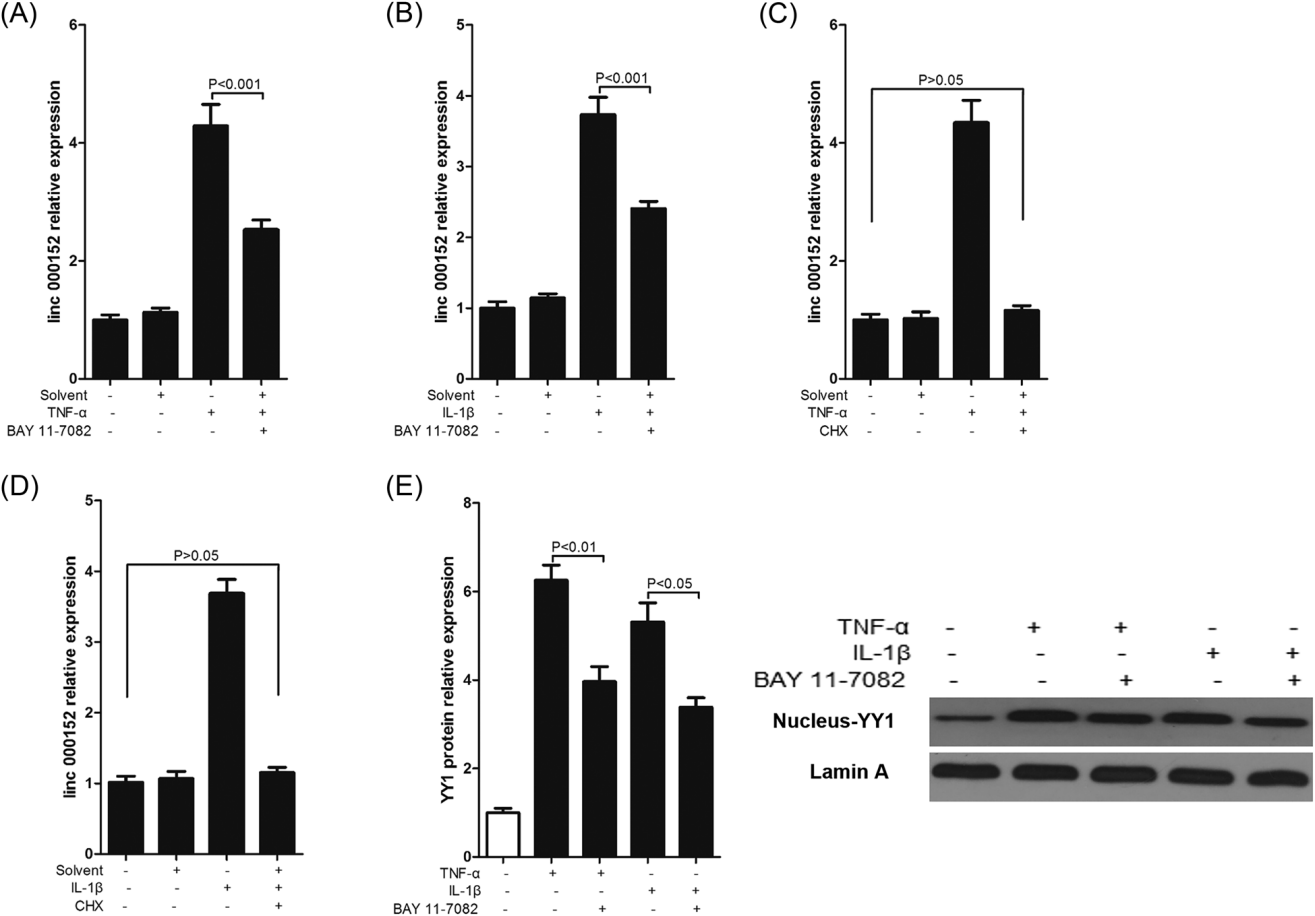
Inflammatory cytokine‐induced increased expression of linc00152 is NF‐kB dependent. RAFLS was first being treated with 5 μmol/L BAY 117082, and then being stimulated with 10 ng/ml TNF‐α (A) or 20 ng/ml IL‐1β (B) for 24 h. At last, we harvested the RAFLS to detect the expression of linc00152. RAFLS was first being treated with 5 μg/ml CHX, and then being stimulated with 10 ng/ml TNF‐α (C) or 20 ng/ml IL‐1β (D) for 24 h. At last, we harvested the RAFLS to detect the expression of linc00152. (E) RAFLS was first being treated with 5 μmol/L BAY 117082, and then being stimulated with 10 ng/ml TNF‐α or 20 ng/ml IL‐1β for 24 h. At last, we harvested the RAFLS to detect the expression of YY1 protein; *p* value was calculated by Student's *t* test. RAFLS, rheumatoid arthritis fibroblast‐like synoviocytes; TNF‐α, tumor necrosis factor‐α

Next, we have a reverse investigation of the effect of NF‐κB on the expression of linc00152. W used BAY 11‐782, an IκBα phosphorylation and NF‐κB inhibitor (BAY 11‐7082 selectively and irreversibly inhibits the TNF‐α‐induced phosphorylation of IκB‐α, and decreases NF‐κB and expression of adhesion molecules.^26^), to inhibit NF‐κB pathway. Here, we found that BAY 11‐7082 can significantly reverse the increased linc00152 induced by TNF‐α (Figure 5A) or IL‐1β (Figure 5B), which indicated that linc0015 and NF‐kB were mutually regulated. To investigate how NF‐kB pathway regulates the expression of linc00152, directly or in directly? We first used CHX, a protein synthesis inhibitor, to inhibit protein synthesis, and then used inflammatory cytokines to treat RAFLS, data showed that after pretreatment with CHX, neither TNF‐α (Figure 5C) nor IL‐1β (Figure 5D) can cause a significant upregulation of linc00152, suggesting that although the downregulation of linc00152 caused by TNF‐α/IL‐1β depends on the NF‐κB pathway, it actually regulates the expression of linc00152 is not the NF‐κB pathway (p65) itself, but a molecule downstream. Next, candidates of putative downstream of NF‐κB pathway were analyzed by previous studies, and we chose YY1 transcription factor as the molecule for further research because not only YY1 was reported to be involved in the regulation of RAFLS inflammation,^19^ but also that YY1 had been found to induce upregulation of lncRNA expression.^27^ Moreover, we retested the regulatory effect of NF‐κB on YY1, and found that BAY 11‐7082 could significantly decrease the expression of YY1 in RAFLS nucleus (Figure 5E).

### 3.6 YY1, a molecule downstream of the NF‐κB, directly promotes linc00152 expression

3.6.1

**Figure 6:**
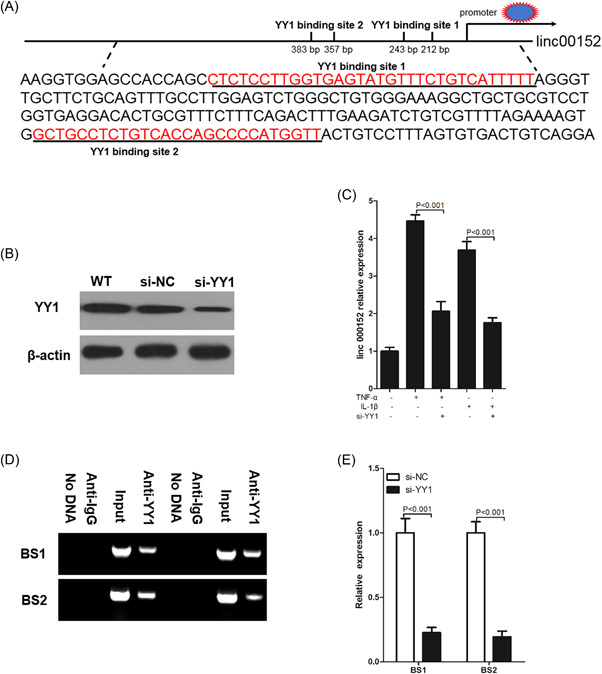
linc00152 is directly upregulated by YY1. (A) The promoter sequence of linc00152, which contains two YY1 binding sites. (B) 72 h after transfection of si‐NC and si‐YY1, we used western blot to determine the expression of YY1 protein in RAFLS. (C) 72 h after transfection of si‐YY1, RAFLS was stimulated with 10 ng/ml TNF‐α or 20 ng/ml IL‐1β for 24 h, and then we harvested the RAFLS to detect the expression of linc00152 using RT‐qPCR. (D, E) HEK293T cells were transfected with siYY1 to reduce YY1 levels, and a ChIP assay was used to detect the direct binding of YY1 to the linc00152 promoter. *p* value was calculated by Student's *t* test. BS1, YY1 binding site 1; BS2, YY1 binding site 2; RAFLS, rheumatoid arthritis fibroblast‐like synoviocytes; RT‐qPCR, real‐time quantitative polymerase chain reaction; TNF‐α, tumor necrosis factor‐α

**Figure 7:**
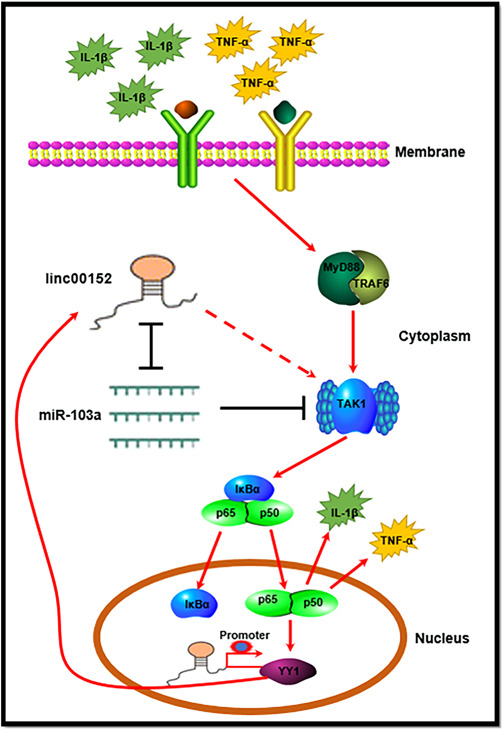
Schematic diagram of linc00152 and NF‐κB pathway feedback loop promotes RAFLS inflammation via regulating miR‐103a/TAK1 axis and YY1 expression. NF‐κB, nuclear factor‐κB; RAFLS, rheumatoid arthritis fibroblast‐like synoviocytes

To investigate the effect of YY1 on the expression of linc00152, we first performed a bioinformatics analysis on the potential promoter region of 2000‐bp upstream of the transcription start site of linc00152, and found that there were two binding sites that bind to YY1 in the 2000‐bp promoter region upstream of the linc00152 transcription start site, namely YY1 binding site 1 (BS1) and YY1 binding site 2 (BS2) (Figure 6A). First, the interfering efficiency of siRNA against YY1 was measured by western blot. Data suggested that si‐YY1 could successfully knock down the expression of YY1 protein in RAFLS (Figure 6B). Next, we studied the effect of YY1 protein expression on linc00152 expression in RAFLS, and found that after being stimulated with 10 ng/mL TNF‐α or 20 ng/ml IL‐1β for 24 h, compared with WT RAFLS, YY1 knockdown could significantly decrease the expression of linc00152 in RAFLS (Figure 6C). To further explore the binding of the linc00152 promoter by YY1 under physiological conditions, a ChIP assay was performed, and the results revealed that YY1 could directly bind the promoter of linc00152 and promoted its expression (Figure 6D,E). Taken together, these results, including the above results, show that inflammatory cytokines (TNF‐α and IL‐1β) promote the transcription activity of YY1 through the TAK1‐mediated NF‐κB pathway, and then promote the expression of linc00152, and linc00152 in turn can promote the expression of TAK1 by binding to miR‐103a, and finally promote the activation of NF‐κB, which forms a linc00152/NF‐κB feedback loop to promote RAFLS inflammation (Figure 7).

## 4 DISCUSSION

RA is an autoimmune disease with symmetric polyarthritis as the main clinical manifestation, characterized by chronic inflammation of the joint synovium and progressive destruction of joints.^1^, ^2^ FLS is a key manufacturer of molecular components in synovial fluid and synovial matrix and plays an important role in maintaining normal joint balance, but it is also the main cellular component in inflammatory synovial tissue of RA.^5^ FLS leads to the erosion of articular cartilage and bone and eventually joint destruction by migrating and invading to the surface of articular cartilage, which plays an important role in the occurrence and development of RA.^5^ In the present study, we first analyzed the microarray data from GEO datasets to obtain differentially expressed lncRNAs, and chose linc00152 as the center of this study. Importantly, linc00152 was not only found to be highly expressed in 11 cases of RAFLS, but also being upregulated by TNF‐α/IL‐1β in a dose‐ and time‐dependent manner in RAFLS. This suggested that linc00152 was inflammation‐related lncRNA and might be involved in the regulation of RAFLS inflammation.

The synovial layer of synovial tissue is anatomically divided into two layers: one layer is the lining layer that is connected to the joint cavity, only 20–40 um thick, and is composed of 1–3 layers of cells loosely distributed on the basement membrane; the other layer is the lower layer of the lining, which is composed of the connective tissue network under the lining layer and the blood vessels and blood cells sparsely distributed therein.^28^, ^29^ There are two main cell types in the lining layer and the lower layer of the lining: FLS and macrophage‐like synoviocytes, and FLS accounts for two‐thirds of them. In the lining layer, RAFLS regulates synovial inflammation by expressing heterophilic linking molecule surface ligands that bind to other cells or extracellular matrix.^30^, ^31^ For example, RAFLS may inhibit the entry of mononuclear cells while allowing leukocytes to enter the joint cavity by expressing vascular endothelial cell adhesion molecule‐1. In this way, it could regulate the ratio of leukocytes to better clean up the debris of the joint cavity and regulate inflammation.^32^ Moreover, RAFLS can produce macrophage stimulating factor and NF‐KB ligand‐activated receptor, two kinds of bone destruction factors, which can erode bone fragments in the absence of osteoclasts.^33^ These findings indicate that the FLS disrupting properties are not only the response of inflammatory mediators to continuous stimuli, but also contribute to RAFLS inflammation. Therefore, studying the molecular mechanism of RAFLS inflammation regulation is of great significance for explore the inflammation of the entire RA synovium.

In this study, the above data indicated that linc00152 was inflammation‐related lncRNA in RAFLS, and the subsequent results showed that linc00152 promoted TNF‐α/IL‐1β induced expression of RAFLS inflammatory cytokines. And the linc00152/miR‐103a/TAK1 axis also be found in RAFLS. Transforming growth factor beta‐activated kinase 1 (TAK1), as a member of the mitogen‐activated protein kinase kinase kinase (MAP3K) family, is a type of serine/threonine enzyme.^34^, ^35^ Previous studies have shown that TAK1 is an important molecule in the NF‐κB‐mediated inflammatory response. TAK1 can be activated by various inflammatory mediators such as TNF‐α and IL‐1β through MyD88/TRAFs, and the activated TAK1 promotes the NF‐kB dimer into the nucleus by degrading IKBα through phosphorylating IKKs to releases.^36^, ^37^ Fortunately, we also found that changing the expression of linc00152 could not affect the expression of MyD88 and TRAF6 protein, two molecules located upstream of TAK1 in NF‐κB pathway, while knocking down linc00152 significantly inhibited the phosphorylation of IκBα and p65, and overexpressing linc00152 promoted the phosphorylation of IκBα and p65. These data suggested that linc00152 activated TAK1‐ mediated NF‐κB pathway by targeting inhibition of miR‐103a in RAFLS.

At the same time, we also found that inhibition of NF‐κB could also inhibit the expression of linc00152 induced by TNF‐α/IL‐1β in RAFLS, and TNF‐α/IL‐1β could not induce the upregulation of linc00152. Because the activation of the NF‐κB signaling pathway does not require the synthesis of nascent proteins, but only the phosphorylation, dephosphorylation and subcellular localization of key proteins have changed.^38^, ^39^ Thus, these data indicated that NF‐κB indirectly regulated linc00152 expression. The transcription factor Ying Yang 1 (YY1) is widely expressed in a variety of tissues, has a complex transcriptional regulation mechanism, and depends on exposure to different protein cofactors to activate or chair related genes. Importantly, YY1 is not only found downstream of the NF‐κB pathway and can be affected by NF‐κB activation, nuclear translocation of p65 can enhance its transcription,^40^, ^41^ but also the previous study have shown that YY1‐regulated LINC00152.^42^ In this study, we found that YY1 promoted the expression of linc00152 by binding to two binding sites in the promoter region upstream of linc00152.

All in all, inflammatory cytokines (TNF‐α and IL‐1β) promote the transcription activity of YY1 through the TAK1‐mediated NF‐κB pathway, and then promote the expression of linc00152, and linc00152 in turn can promote the expression of TAK1 by binding to miR‐103a, and finally promote the activation of NF‐κB, which forms a linc00152/NF‐κB feedback loop to promote RAFLS inflammation. Thus, linc00152 acts as a switch to control this regulatory circuit and may serve as a diagnostic and therapeutic target for RA treatment.

## Data Availability

The data that support the findings of this study are available on request from the corresponding author.
